# Multidisciplinary teams and ICT: a qualitative study exploring the use of technology and its impact on multidisciplinary team meetings

**DOI:** 10.1186/s12913-018-3242-3

**Published:** 2018-06-13

**Authors:** Anna Janssen, Tracy Robinson, Melissa Brunner, Paul Harnett, Kylie E. Museth, Tim Shaw

**Affiliations:** 1 Faculty of Health Sciences, Charles Perkins Centre, The University of Sydney, Level 2, Charles Perkins Centre D17, Camperdown, NSW Australia; 2 Sydney West Translational Cancer Research Centre, Westmead Hospital, Westmead, NSW Australia; 3 Monash Centre for Health Research and Implementation, School of Public Health and Preventive Medicine, Monash University, Melbourne, Australia; 4 Faculty of Education and Arts, The University of Newcastle, Callaghan, NSW Australia; 5 Crown Princess Mary Cancer Centre, Westmead Hospital, Westmead, NSW Australia

**Keywords:** Multidisciplinary care, Cancer, Patient-centered care, Data, Technology

## Abstract

**Background:**

Multidisciplinary teams (MDTs) are an integral component in the delivery of health care. This is particularly evident in the delivery of cancer care, where multidisciplinary teams are internationally recognized as the preferred method for service delivery. The use of health information systems and technology are key enabling factors for building the capacity of MDTs to engage in improvement and implementation projects but there is scant research on how MDTs make use of technology and information systems or the kinds of systems needed for them to undertake improvement and implementation research. This paper reports findings on how seven MDTs in cancer care utilized technological and information systems and the barriers and enabling factors that impacted on their uptake.

**Methods:**

Seven multidisciplinary teams from two large metropolitan hospitals participated in the study. Qualitative methods including structured observations and semi structured interviews that explored how teams engaged in research and improvement activities were utilized. Participants were also observed and interviewed in relation to their use of data and health information systems. Findings were subject to content analysis and key themes were identified. Interviews were transcribed and de-identified and key themes were subsequently discussed with participants to allow for member checking and further clarification of findings.

**Results:**

A total of 43 MDT meetings across seven tumor streams were observed. Of these, observation notes from 13 meetings contained direct references to emerging technologies and health information systems. Findings from 15 semi-structured interviews were also analyzed in relation to how MDTs used technology in weekly meetings, and the perceived impact of technology. Three broad themes emerged: (1) methods for data collection and use by MDTs, (2) the impact of technology on the MDT meeting environment, and (3) the impact of technology and information systems on clinical decision making.

**Conclusion:**

The study demonstrates that real time data collection and imaging may improve patient centered care coordination. However, ICTs can be used sub-optimally by teams. We therefore urge additional research to identify the enabling factors that support better collection and use of outcome data from ICT.

**Electronic supplementary material:**

The online version of this article (10.1186/s12913-018-3242-3) contains supplementary material, which is available to authorized users.

## Background

An increasing body of literature is focused on investigating how existing and emerging information communication technologies (ICT) are impacting the health sector, both for patients and health care professionals. The literature reveals ICT is rapidly changing the way individuals engage with their own health [1]. Additionally, research shows ICT is improving clinical processes by supporting data-driven care and allows patient outcomes to be measured and compared to benchmark performance metrics [2]. Finally, the contemporary data revolution transforming the health sector is being driven by improvements in ICTs [3]. This is because advances in technology make health data accessible and actionable, enabling clinicians to change their practices [4]. It is clear from the literature that the widespread use of existing and emerging technologies are reshaping healthcare. However, there are notable gaps in understanding how technology can be used to better understand the patient experience and how technology in areas such as cancer care can be utilized to improve patient-centered models of care. There is a recognized lack of understanding of how ICTs can be used to support the delivery of quality care for patients, and enable linkages across different applications and data sets [5]. Furthermore, the literature that uptake of ICT in the health sector is more common when the functionality of the technology offers financial benefits, then whether they offer quality or safety benefits [6].

A decade ago it was identified in the literature that ICT systems could be of benefit in health care due to their capacity to capture rich patient data and support standardization of care [6]. In addition, real time decision support for oncologists and service users, has the potential to significantly improve clinical outcomes [7]. Despite a consensus that the use of ICT systems should lead to more efficient and high quality care [8] the integration of these systems into cancer services has been slow and uneven. Overall, there is a paucity of literature that addresses how technology can be integrated effectively into cancer care. One significant barrier to the successful implementation of ICT is the perception that newer systems such as electronic medical records and databases offer no significant improvement over existing approaches such as paper-based notes, and in many instances may increase the workload of clinical staff [9]. Other cited challenges include questions about the security and reliability of web based information, uneven access to online facilities and a lack of operator skills among health workers [6]. There is evidence in the literature demonstrating that ICT can be used to improve data collection, but this is often limited to specific medical disciplines such as surgery [10]. However, there is also evidence to suggest that poorly implemented ICT in healthcare can contribute to the wide scale collection of poor quality data [11]. One of the major challenges to overcome in the implementation of ICT for data collection is improving linkages between systems and data access issues [12]. In addition to this, despite collecting a plethora of performance indicator data, meaningful measures of patient experience, equity and population health outcomes remain elusive [13]. The use of ICTs in cancer care is an important issue, which warrants further exploration.

A core component in the delivery of cancer care is the use of multidisciplinary teams (MDTs). The central function of MDT meetings is to bring together a team of health care professionals to determine a patient’s treatment plan [14]. MDT meetings are widely used in the health care sector and in the UK they have been recognized at a policy level as the preferred means of delivering care [15]. MDTs are not just central to the delivery of cancer care, but are a central component in the delivery of healthcare more generally. This is particularly evident in the literature on the benefits of multidisciplinary care to create links between specialists and primary care providers [16]. The literature has demonstrated that team-based approaches to healthcare delivery can improve some aspects of the quality of care delivered to patients [17]. Furthermore, team-based care can benefit health professionals as well as patients, by improving understanding of patients care plans and overall professional climate [18].

Although MDTs are a central component of cancer care in many countries, there is a notable gap regarding how technology and information systems can be integrated into these teams. The lack of research into how ICTs can be integrated into MDTs is concerning considering the importance of well implemented ICTs on the coordination of care by health professionals [2, 19]. The use of ICTs in the form of clinical dashboards and clinical decision support systems have also been shown to improve processes and patient outcomes for cardiovascular multidisciplinary teams [20]. Further, the literature has demonstrated ICTs used to facilitate multidisciplinary blood pressure management between treatment teams and patients can improve blood pressure management and control [21]. Finally, a recent review of the literature concluded that successful implementation of ICTs in multidisciplinary care has the capacity to enable universally accessible, cost-effective, and high quality care, particularly for patients in rural and remote locations [22].

In a previous paper, the authors reported findings from an ethnographic study on the engagement of MDTs in translational research and quality improvement [23]. One of the findings of this study was that the capture of real time data was a priority to help engage teams more actively in quality improvement activities [23]. There is also evidence in the literature that the use of specific ICTs, such as clinical support technologies, have potential to enhance a range of aspects of MDTs including operational and governance aspects [24]. However, effective use of innovations such as disruptive ICTs is more complex than just making them available, and also requires the use of additional solutions such as promotion of a culture of innovation in, access to real-world data and implementation of a whole-system approach to guide investment in innovation [25]. This highlights the need to better understand the use of ICT systems in cancer care and the specific capabilities needed to support integrated health information.

The current literature on the use of ICT in MDT meetings indicates its use varies according to a range of contextual factors, including the culture of MDTs and the meeting environment, that have been shown to have a significant impact on how effectively a team can use and access technology [26]. MDT meetings often take place in clinical spaces that are not built to facilitate interaction with technology and where the presentation of patient imaging is challenging and access to high-speed internet is not guaranteed [15]. There is very little research into the uptake and usage of ICTs in clinical teams like MDTs, and how they impact on team dynamics. Increasing understanding of the use of technologies is a key component in improving the implementation of current and future technologies to enhance the capacity of MDTs to lead healthcare improvement and innovation. This is particularly important in relation to the growing prevalence of ICT such as electronic medical records (EMRs) which are transforming the collection and redistribution of clinical data [8]. However, maximizing the use of EMR data by MDTs is not just about improving collection but understanding how teams can best make use of it to lead healthcare improvement and innovation.

The project described in this article was part of a wider research study, exploring the barriers and enabling factors that support research translation and implementation projects in oncology MDTs. This article presents findings on the specific use of ICTs in an MDT program of cancer care at two sites in Sydney. The primary objective of the study presented in this article was to identify how oncology MDTs currently used ICTs, and to explore how the use of these ICTs affected interactions within MDT meetings. The study was undertaken prior to the scheduled progressive roll out of Oncology Management Information Systems across Australian hospitals.

## Methods

This paper reports secondary findings from an ethnographic study on the engagement and use of translational research and quality improvement by MDTs in cancer care [23]. Data were collected from unstructured observations and semi-structured interviews with members of seven MDT tumor stream meetings at two sites in Sydney in 2014. MDTs self-selected to participate in the study and included the following streams: Breast, Upper Gastrointestinal, Lower Gastrointestinal, Lung, Gynecological Oncology, Metastatic Breast, and Melanoma meetings.

Three researchers (TR, AJ, KM) conducted unstructured observations of participating MDTs over a period of 2 months. Each researcher took field notes during the MDT meetings and recorded their observations of the meetings. Analyses of observation notes involved identifying all references to the theme of health information systems and technology and how it was used. Comments were grouped according to broad themes that provided information on contextual factors such as how information systems were used in meetings and whether these systems supported treatment decisions.

At the conclusion of the observation period, semi-structured interviews were conducted with individual MDT members. The semi-structured interviews focused on exploring themes around the use of health information systems and technologies that had emerged from analysis of the observational notes, in order to explore them in more depth [27]. Semi-structured interviews took between 30 to 60 min and were conducted face-to-face. Refer to Additional file 1 to see the semi-structured interview schema. Interviews were recorded, transcribed, and de-identified. The interview transcripts underwent an initial reading to identify terms that indicated the use of technology or ICTs. The researchers discussed the terms in the text and agreed on how they could be grouped into broad categories. The researchers then discussed the context of keywords in the transcripts and reached a consensus regarding which broad category would be most appropriate for the keyword. These broad categories and keywords were translated into a reference table for use in subsequent readings, and structured categorisation of the transcripts. (Table 1 *identifies broad categories identified by consensus and aligning key words).* Subsequent readings of the transcripts were informed by the broad categories and keyword in the reference table, and enabled a structured review of the transcript to identify themes that were consistent across interviews [28].

**Table 1:** The final reference table used to conduct a structured review of the interview transcripts. The broad categories, and the keywords the aligned with them were determined by consensus between the review team

Broad categories	Keywords
Mobile devices	Mobile
	Cell
	iPhone
	Android
	Phone
	Pager
	Beeper
General technology	Technology
	Electronic
	Digital
Hardware	Computer
	Laptop
	Screen
	Projector
	Microscope
	Light box
Software	Internet
	Online
	Google
	Database
	Data
	Search engine
Imaging	Xray
	Scan
	ECG
	Pathology
	CT
	MRI
	Imaging
	Testing

Permission to conduct this study was granted by human research ethics committees of the University of Sydney, the local health district.

## Results

### Results from structured observations

A total of 43 MDT meetings (MDTMs) were observed and their notes reviewed. Of these, a sub-set of notes from 13 MDTMs contained direct references to how the teams used technology and the impact its use had on the MDTM. Every location had facilities to project images onto a screen, as well as access to at least one computer. Some locations also had access to other technologies such as televisions, imaging equipment, teleconferencing facilities, lightboxes, and microscopes. Only two of the meetings had dedicated administrative support*.*

Irrespective of location, all meetings had an established seating routine, consisting of an inner and an outer circle of members. The small size of the room often reinforced this seating arrangement with a large table occupying most of the space. The inner circle consisted of senior clinicians including, oncologists, surgeons, radiologists, senior nursing staff and pathologists. The outer circle consisted of junior doctors and trainees, medical students, allied health, clinical trial nurses, and late attendees. The researchers observing the meeting also sat in the outer circle. The location of hardware dictated that imaging staff sat in the outer circle of the room. In MDTM rooms screens, microscopes, light boxers and desktop computers were located at the edges of the room, with a large table in the central space.

Overall, technology was most frequently used to display patient imaging. Screens at the front of the meeting room displayed patient scans and imaging experts reported their interpretation of the results. The display of patient imaging and scans was a clear facilitator of discussion across specialties, particularly between oncologists and pathologists. It was also often a source of discussion between the inner and outer circles of the meeting as more senior specialists asked questions of junior doctors seated in the outer circle in relation to treatment options and prognosis. In one MDT tumor stream meeting the chair regularly engaged junior doctors in the outer circle by asking them to conduct internet searches for literature to support certain diagnoses.

Challenges in relation to relying on imaging equipment were also evident. On occasions imaging or imaging reports were not available or older images could not be accessed to allow team members to assess disease progression. This was a result of poor access to patient databases or gaps in the available data. Imaging was observed to be unavailable due to either to the challenge of integrating digital systems or because results had not arrived from external imaging providers. A dedicated meeting coordinator was observed to play an important role ensuring timely access to imaging. This meeting coordinator ensured that agendas, patient lists and reminders were disseminated and this helped ensure all relevant results were available for the MDTM. At those meetings that had dedicated administrative support agendas, patient lists and reminders were disseminated and this helped ensure all relevant results were available.

One MDT used teleconferencing equipment to support a satellite MDT in a rural location. Despite having the capacity to link with rural areas this was generally only used as required or when requested by a clinician. Another MDT encouraged junior doctors to deliver a PowerPoint presentation of a clinical case study they had encountered for discussion with the team. This MDT also allowed time for research-focused members to present their research. Another MDTM chair encouraged registrars to regularly use smartphones to source information on treatment options and diagnosis. Only one team entered ‘real time’ data during the meeting and this was led by a registrar who was undertaking a local healthcare improvement project.

Overall, findings from observations confirmed the importance of imaging equipment to diagnose and discuss treatment options. The use of imaging impacted on the environment of meetings and stimulated team discussion. Only one team utilized information systems to enter ‘real time’ data and one used teleconferencing facilities.

### Semi structured interview results

A total of 15 interview transcripts contained references to technology, how MDTs used technology in their weekly meetings, and key enabling factors for improved use of health information systems. Three broad themes emerged, including the use of technology for data collection, how environmental factors and the use of health technology in MDT meetings technology shapes the MDTM environment, and the impact of technology on clinical decision making. Table 2 contains exemplar quotes for these themes *(Refer to* Table 2 *to review illustrative quotes categorized by theme****).***

**Table 2:** Semi-structured interview illustrative quotes categorized by theme

Theme	Illustrative Quotations
Theme 1: Data collection	*“I think data collection is of the upmost importance. That is number one. And if we can get a better way to record our data as opposed to our paper files then fantastic.”*
	*“The best way really, for collecting this sort of data would be to be filling it out at the MDT.”*
	*“What I have noticed is that people are obsessed with having their own data, I got data through my MDT”*
	*“A lot of MDTs have their own database that they have had for many years but it’s not linked to anything so it’s actually not useful data. Unless you can link it with something and did you use it for anything, what’s the point of collecting data?”*
	*“Any data that we collect should be meaningful. I mean, we collect data all over the shop, but it doesn’t mean to say that we’re getting it fed back to us in the right format…why are we doing audits on patient calls and why are we doing medication audits? It means nothing to them [clinicians] in that format. So I think it’s about just making it [data] meaningful for the clinician.”*
	*“If you can get the data and give them sensible data, you’ll get improvement. But getting the data, and getting the data that - I think it’s going to be really important to get only a few bits of data and get the data that the key stakeholders really want and need. Two types of data: times, time from symptoms to presentation, time from presentation to referral, time from referral to the MDT, time from referral to definitive management or MDT to definitive management. Outcomes, which is probably just length of life at the moment, but if there’s other outcomes as well.”*
Theme 2: Environmental factors and the use of health technology in MDT meetings	*“It’s been a little bit rocky at the moment. There is some conservatism among some clinicians about how they prefer to do these discussions. It’s going to be a little slow to impose a little bit more order on it but unless we do we can’t improve our MDT process.”*
	*“Part of the problem is there has been a project to try and develop an electronic medical record for the past seven years and there has been lots of problems and setbacks. It hasn’t happened so you’re kind of living between two half worlds and neither record is complete. You’ve got some stuff electronically, some in paper. It can often take you 20 min if you’re seeing a patient to make sure you have all the right information.”*
	*“In the subset of patients who increasingly now are having complex molecular testing where we brought in a modification of the MDT process with some standard data presentation slides and a pre meeting to discuss them. It’s different from what people are used to so even in this very savvy group my hope was that they would see the benefits of having a structured visual supported. Some basic templates where the information is always in the same format, they just get used to seeing that, they can rapidly simulate information. If it works for this molecular data, it would work for the standard patient.”*
	*“we’re big on the electronic, doing things in real time electronically. What I have been advocating when I’m going out there is for those who use Mosaic or whatever to actually have it up on a screen typing in real time in not just free text but in something that can then take that information and do your GP letter, a* pro forma *GP letter, can do a GP plan, patient letter for you and then can do the onus extract into the registry.”*
	*“I think something integrated [with the MDT] would be good, because I think if it’s integrated and you know what data you’re collecting, and if you can provide feedback to them, you’ll be on a winner. So you know, once a month at the MDT, spend fifteen minutes, get up there and say, look, last month this is what happened with the patients.”*
	*“We can collect a lot more data at the MDT... Once we have the MDT database up and running I would want the clinicians to be more involved in entering data in the meeting. We want the clinician who is presenting it ... One physician finished presenting another physician should be seeing another case and then another clinician can update everything for the MDT database and that would be accurate. Then we just rotate through instead of one-person keeping on talking. That would help a lot I think.”*
Theme 3: The impact of technology on organizational processes	*“The other thing is, in the MDT, if you haven’t booked it in they won’t have the images there. And it’s so irritating.”*

#### Theme 1: Data collection

Participants were unanimous that data collection was important and enabled by health information systems. Several also commented on the implementation of an EMR and stated that it would only be beneficial if it contained accurate, high quality data. They expressed concerns about the quality of data that was currently collected through MDTMs. One participant reported that patient-centered data such as quality of life was noticeably absent and that this was a significant gap in understanding how treatments affected patients and their discussions around end of life care.

Several barriers to the collection of quality data were identified, including that it was time consuming to collect, that there was duplication across multiple databases which could lead to errors, or that there was no one in the MDTM to collect the data. One participant felt that the quality of data collection varied across disciplines, as areas such as radiology and surgery had specific reporting requirements that effectively mandated the collection of specific data. Another noted that the collection of routine data such as length of stay or time between admissions was not indicative of treatment outcomes or quality of life. Ultimately, data collected from the MDTMs varied according to the skills of individual members and the resourcing of individual teams. Nevertheless, all participants identified that information systems should be used to collect high quality data and that MDTMs present a unique opportunity for improving data collection.

Participants also commented on the burgeoning number and duplication of databases and the challenges this posed in terms of data linkage and ownership of data. Two interviewees noted a reluctance by clinicians and researchers to share their data, which could reflect legal and confidentiality concerns. Reticence to share data was considered as a barrier to promoting evidence-based care. This kind of ‘territorial’ approach to data was also seen as one of the reasons for multiple and replicated databases. The proliferation of duplicate databases is problematic because they are usually unlinked and not contextualized within a larger body of either local or global population data.

Using innovative technologies such as linked databases that allowed real time data entry and analysis was considered challenging. It was reported that health information systems were not always implemented well and were poorly integrated into MDTMs. This, in turn, fueled resistance to new systems. The importance of time and support were highlighted to avoid data collection impacting on clinical workloads and most participants recommended more resourcing for MDTs in relation to this. In particular, funding for a dedicated data manager who understands the structure of clinical databases and who could attend MDTMs regularly was identified as a key enabling factor. Participants reported that ideally, the same person who collected the data would enter it into the database and suggested that the MDTM could be used as a quality control opportunity to review the data and ensure its accuracy. One disparate comment related to utilizing technology to feed data back to MDTMs and noted that this would allow clinicians to engage with their data and better identify quality gaps and improvement issues.

#### Theme 2: Environmental factors and the use of health technology in MDT meetings

A number of participants referred to cultural and environmental factors that influenced the use of information systems and imaging equipment within meetings. The ability to display and interpret patient scans relied on an appropriate meeting room and the attendance of specialists such as pathologists who have the ability to interpret the images. In one MDT, the pathologist did not always attend and delays occurred when their opinion was required. This highlights how the effective use of technology relies on both location and clinician expertise.

It was identified that common electronic tools had the capacity to shape and improve MDTMs. Digital tools such as PowerPoint slides were easily available to present patient case reviews at MDTMs, but were rarely used. The need to improve the MDT environment to create a vehicle for collecting good clinical data was discussed extensively as was the importance of having members with specialized knowledge such as programming to ensure the optimal use of technology. However, it was felt the current system made access to these experts difficult.

#### Theme 3: The impact of technology on organizational processes

Participants were unanimous that health information systems and imaging had an important role in supporting clinical decision making. This was supported by observations that confirmed the role of imaging in facilitating discussions about disease staging, treatment, and prognosis among team members. When patient scans were unavailable delays in treatment decisions occurred and one participant stated there was a need to identify which external imaging companies made scan results available online, and that patients should be referred to only those companies for their scans to ensure access to timely treatment. Timely access to imaging was crucial for helping to identify discrepancies in previous diagnoses and enhancing the ability to compare and discuss individual treatment plans. Additionally, all participants agreed that imaging technology and real time data supported and enhanced clinical discussion during meetings.

## Discussion

The findings from this study indicate that MDTs currently use a wide variety of ICT in support of and during their meetings. This aligns with the broader literature which indicates that there is widespread use of technology in the health sector and that it is transforming the delivery of care [1, 2]. ICTs have great potential for improving aspects of healthcare, and they have been used in innovative ways in the health sector [3]. However evidence on the effectiveness of these innovations is lacking, and there are a range of challenges still to be overcome [24, 29]. Findings from this study build on the existing literature by suggesting that MDTs currently do not make optimal use of health information systems and technology. There are several barriers to optimal use of ICT during MDT meetings including, physical environment, time, and ICT skills of individual team members. The barriers identified in this study are consistent with the perception that new technologies are burdensome and increase workloads [30].

Although this study focused broadly on the use of ICT in MDTs, participants identified the collection and use of real time clinical data as a priority. In addition, MDT leaders and champions expressed a desire to collect meaningful patient-centered data (such as quality of life) that could help inform treatment choices and end of life care. This is consistent with findings that acute and long term physical and psychosocial comorbidities are associated with cancer treatment, indicating there is an increasing need for supported self-management and shared care [9]. Furthermore, patient experience or patient reported outcome measures are an indicator of service quality [31]. MDTs have an important role in this process and if we are to improve treatment decisions and quality of care we need to identify the key outcomes and data relating to patient experience that can inform this process. Well implemented ICTs for data collection have the potential to improve the coordination of cancer care in MDTs, as they have been shown to do in other health areas [21].

Overall, participants felt ICT had the potential to make clinical data collection more accurate, and easier to feed back to MDTs for a range of applications. This finding aligns with clinical data research, which has shown that the increasing use of technologies such as electronic medical records has made data more accessible to clinicians by opening up opportunities to access their data for a range of uses from clinical processes to quality improvement [29, 32]. At the same time, some participants expressed their concern that health services now collect a plethora of data but much of it lacks utility and accessibility. The literature has demonstrated that the widespread use of ICTs for clinical data collection has had the effect of collecting vast amounts of inaccurate or unnecessary data [11]. Findings from this study showed clinicians themselves are aware of the risk of collecting data that lacks value. Moving forward, the challenges in identifying which clinical data to collect may be overcome through the engagement of end-users, such as health professionals, in the design and implementation of data collection ICTs.

Although ICTs can facilitate the collection of unnecessary data, findings from this study indicated that technology has the potential to be an enabling factor in improving the collection of high quality clinical data, as it can make data input straightforward and easier to feed back to clinical teams. Technology that enabled efficient collection of clinical data was demonstrated by one participating MDT that used technology to incorporate ‘real time’ data collection into their meetings. Interviews with participants of this team identified that not only was this more efficient, but also the team collection of meaningful, patient-centered data was helpful for informing clinical decision making and end of life care. This aligns with previous research that identified ‘close to the source’ data increased the quality of data collection [30]. Hence, data collection may be considered a dimension of service quality and used as an indicator of quality. MDTs have an important role in this process and if we are to improve treatment decisions and follow up care we need to identify the key outcomes and data that can inform this process.

An integral part of this process will be ensuring that clinicians, themselves, see the value and feasibility of collecting patient-reported outcomes (PROMs). ICTs have the potential to integrate PROMs into routine data collection systems and more research into how this can be enabled in cancer care is imperative [33]. Indeed, there is an urgent need to identify a taxonomy of core PROM domains and dimensions in cancer because these are rarely implemented in clinical practice despite wide acknowledgement that it is crucial to capture the patient’s experience of treatment and care [31].

In spite of the interest in the use of ICTs to improve aspects of the MDT such as data collection, findings from this study suggest the use of ICT in and of itself is insufficient in changing team dynamics and improving the delivery of care. ICT on its own is a tool that can be harnessed to enhance MDTs, but there are also human and organizational factors that support successful use of technologies in health care. A particularly important one for MDTs is having access to specialists who understand how to use and interpret the technologies and information systems, such as experts who can design clinical databases for teams or analyze clinical data sets during meetings. Thus the successful implementation of health technologies may require task re-allocation in order to ensure relevant staff have adequate skills to use the technologies optimally [8, 34]. Furthermore, complex technologies often require organizations to invest in individuals to ensure they possess skills with specific technologies in order to support their implementation in MDTs [35]. The need to upskill existing staff or engage staff with specialised ICT skills may increase costs for healthcare organisations in order to ensure ICT sustainability. Such an outcome stands at odds with much of the currently literature which champions ICTs as a cost-effective solution for improving the quality of healthcare [22].

Although the literature acknowledges the benefits of multidisciplinary care for both patients and health professionals, there are still gaps in understanding as to how best support this type of care [24]. This lack of understanding is coupled with the challenges of implementing innovations in MDTs, such as adequate uptake and cost-effectiveness [25]. Findings from this study indicate that in order to overcome implementation challenges for innovations such as ICT within MDTs it is important for both individuals and organizations to recognize when and how it can be used to benefit practice. This is particularly important in rapidly evolving areas such as the collection and feedback of data for both clinical and research purposes. Doing this effectively requires information technology experts, implementation researchers, and clinical teams to work together to develop implementation approaches for using ICT in a way that will lead to behavior change. Organizations will also need to acknowledge and support the need for resourcing in order to utilize emerging technologies in optimal ways.

### Limitations

This research study asked two questions how MDTs use ICTs and how this impacted on interactions in meetings. In regards to answering the first question, the study is limited by the fact that it only represents the observations of MDT members at two sites, over a sampling of MDTMs. Given that the MDT members were aware they were being observed, it is possible they were affected by the Hawthorn effect. The Hawthorn effect states that all observational studies are vulnerable to the Hawthorn effect whereby participants alter their behavior in response to being observed. The researchers attempted to overcome this limitation by attending multiple meetings of each tumor stream over the course of the study, and attending a MDTMs across a broad range of tumour streams. It must also be acknowledged that data was collected through unstructured observations. As such, it is possible that only a small sub-set of observational notes recorded ICT use due to variation in what the observers recorded. Finally, the study observations focused on an area of practice that is experiencing rapid change: ICT usage, at a specific moment of time, as a result findings cannot be generalized across settings. Additionally, elements of ICT observed in the organisation when this study was undertaken may have changed during the publication process. In regards to answering the second question the study is limited by fact that it relied on consensus of researchers to identify categories and themes as such, categorisation of the data is potentially limited by the understanding of the researchers of the definitions of the categories and keywords.

The project reports on two phenomena in order to get a comprehensive picture on the use of ICTs in MDTs: 1) Interactions of MDTs and 2) the use of ICT by MDTs. As two phenomena are being observed, confounding factors make it difficult to draw clear conclusions as to which of the two phenomena are creating the result. However, confounding factors are a common barrier in implementation and health services research and it is still beneficial to observe the way ICTs may alter MDTM interactions.

Although the methodology used in this research project was beneficial for understanding how oncology MDTs currently used ICTs and how the use of these ICTs effected interactions within MDT meetings, alternatives could be adopted by future researchers. This could include focusing observations on an individual tumour stream to minimise variation of observations across specialties. Alternatively, researchers could observe MDT meetings during the same period (ie 4 weeks at the start of a calendar year) over multiple years to understand how ICT use is integrated and sustained over time.

## Conclusion

The use of ICTs is commonplace in MDT meetings, particularly technologies for displaying patient imaging and other pertinent patient history information. However, ICTs continue to be underutilised for real time data collection and feedback, an application that is of particular use to health professionals. Improving real time data collection and feedback has the potential to improve quality, care coordination and patient-centered models of care. This potential is not always realized and there is value in identifying the enabling factors that support better collection and use of outcome data and other health information systems. Although there are a range of barriers to implementing ICT effectively in healthcare, there are also a number of factors that may increase the likelihood of successful integration. From an organisational perspective, it is important that adequate resourcing is provided so that MDTs have access to the right technologies at the right time and place. From the perspective of MDTs effective implementation of ICTs can involve engaging members who have the skills to use specific technologies optimally, so that technologies can be harnessed in a way that is synergistic with the existing structure of the MDTM.

## Additional file

Additional file 1: Semi structured interview schema. The semi-structured interview prompts used to inform the interviews with MDT members. (DOC 28 kb)

## Abbreviations

CSP: Cultural systems paradigm
EMR: Electronic medical record/s
MDT: Multidisciplinary team
MDTM: Multidisciplinary team meeting
