# Supplementary material for: Multidisciplinary teams and ICT: a qualitative study exploring the use of technology and its impact on multidisciplinary team meetings
Source: BMC Health Serv Res. 2018 Jun 13;18:444. doi: 10.1186/s12913-018-3242-3 (PMC6001028; doi:10.1186/s12913-018-3242-3)
Supplement: Supplementary file 1 — Semi structured interview schema. The semi-structured interview prompts used to inform the interviews with MDT members. (DOC 28 kb) [file 12913_2018_3242_MOESM1_ESM.doc]

**Semi-structured Interview Questions**

1. Could you please describe the MDTs you are currently involved in?
2. How many regular members are currently in your MDT?
3. Does your MDT have a mission statement and Terms of Reference? If so, do they address research and education?
4. Is there a formal structure for determining eligibility for MDT membership? If so, please specify what it is?
5. Is there a formal leadership structure for the MDT? If so, how it is determined?
6. How frequently does your MDT meet for formal meetings?
7. Do MDTs meet for any purpose other than case review? If so, how frequently?
8. What organisational support is available to enhance the role of MDTs across the spectrum of health service delivery?
9. To what extent does your MDT use ICTs in your meetings?
10. Does your MDT have any system for collecting clinical data in your meeting?
11. X was observed during MDT meetings, could you please discuss how X effects MDT interactions *[Insert X based on analysis of observations].*
